# Fibrillar Hydrogel Based on Cellulose Nanocrystals Crosslinked via Diels–Alder Reaction: Preparation and pH-Sensitive Release of Benzocaine

**DOI:** 10.3390/polym15244689

**Published:** 2023-12-13

**Authors:** Sofia M. Morozova, Evgenia G. Korzhikova-Vlakh

**Affiliations:** 1 Center of Fluid Physics and Soft Matter, N.E. Bauman Moscow State Technical University, 2nd Baumanskaya St. 5/1, 105005 Moscow, Russia; 2 Institute of Macromolecular Compounds of Russian Academy of Sciences, Bolshoy pr. 31, 199004 St. Petersburg, Russia; vlakh@hq.macro.ru

**Keywords:** cellulose nanocrystals, Diels–Alder reaction, aldehyde cellulose nanocrystals, benzocaine, pH-sensitive release

## Abstract

A fibrillar hydrogel was obtained by covalent crosslinking via Diels–Alder reaction of two types of cellulose nanocrystals (CNCs) with furan and maleimide groups. Gelation has been studied at various ratios of components and temperatures in the range from 20 to 60 °C. It was shown that the rheological properties of the hydrogel can be optimized by varying the concentration and ratio of components. Due to the rigid structure of the CNCs, the hydrogel could be formed at a concentration of at least 5 wt%; however, it almost does not swell either in water with pH 5 or 7 or in the HBSS buffer. The introduction of aldehyde groups into the CNCs allows for the conjugation of physiologically active molecules containing primary amino groups due to the formation of imine bonds. Here, we used benzocaine as a model drug for conjugation with CNC hydrogel. The resulting drug-conjugated hydrogel demonstrated the stability of formulation at pH 7 and a pH-sensitive release of benzocaine due to the accelerated hydrolytic cleavage of the imine bond at pH < 7. The developed drug-conjugated hydrogel is promising as wound dressings for local anesthesia.

## 1. Introduction

Hydrogels based on cellulose nanocrystals (CNC) are very promising for various biological applications due to their biocompatibility, lack of toxicity and fibrillar structure [1,2]. Hydrogels based on CNC can be formed by both weak physical interactions [3,4,5] and covalent crosslinking [6,7,8,9]. However, physically crosslinked hydrogels often break down over time due to their dissolution in the environmental liquid medium as a diluent [10], which focuses their potential application on biodegradable implants or drug delivery systems [11], while for patches, wound dressings and stabile drug delivery carriers, it is preferable to use covalently crosslinked gels that retain their shape and are not subject to degradation [12]. For the aforementioned application of covalently crosslinked hydrogels, a number of requirements are presented to them, including uniformity, bioorthogonality of the crosslinking reaction and minimization of by-products and catalysts used to maximize biocompatibility [13]. For example, covalently crosslinked hydrogels may be characterized by heterogeneity, for example, if they are crosslinked too quickly [14], or if the gel is obtained under the influence of an external stimulus, such as, for example, UV-irradiation [15], which is also distributed inhomogeneously in the sample. Therefore, it is preferable to use a reaction that does not proceed instantly and does not depend on external radiation or chemical gradient.

The Diels–Alder reaction fully complies with these requirements, and, since it is bioorthogonal, does not require activation by additional chemicals, unlike, for example, UV or thermo-induced free radical polymerization, and no by-products are formed when the components interact [16]. The Diels–Alder reaction consists of the interaction of the diene component and the dienophile with the formation of new C-C bonds [17]. The most commonly used diene–dienophile pair for hydrogel fabrication is the furan–maleimide functional groups [18,19,20,21]. Most hydrogels based on this reaction are created using polymers such as hyaluronic acid [22], chitosan [23], gelatin [24] and PEG [25] functionalized by furan and maleimide groups. Examples of using CNC as one of the hydrogel components are limited to two reports in which the CNC system was used with furan-modified gelatin [9] or maleimide terminated PEG [8]. At the same time, in both examples, the polymer/CNC ratio did not allow for achieving the fibrillar structure of the gel, while the fibrillar structure mimics the structure of many living tissues [26].

Of particular interest are hydrogels capable of programmable release of active substances, i.e., in which the release can be regulated by an external stimulus, such as a change in pH [27], temperature [28] or ionic strength [29]. The pH-mediated drug release is promising for the treatment of many pathological processes associated with a decrease in the physiological pH of the environment. For example, acidification is characteristic of open wounds during infection or chronic wounds [30], some diseases of the gastrointestinal tract [31] as well as tumor tissues [32]. The most well-studied component of pH-sensitive gels is chitosan [33], which has a disadvantage associated with its significant swelling at low pH. The development of a pH-sensitive gel with minimal volume changes is promising for the development of programmable wound dressings and patches.

Here, we present a nanofibrillar hydrogel consisting of two types of cellulose nanocrystals, with furan (Fur) and maleimide (Mal) groups, covalently crosslinked via Diels–Alder reactions. By changing the composition of the hydrogel, it is possible to regulate its mechanical properties. In addition, an introduction of aldehyde groups into the structure of the components allows the formation of pH-sensitive imine bonds with physiologically active substances containing amino group(s). The key property of the final hydrogel delivery system is its ability to stimulus-sensitive drug release without destroying the structure of the gel itself, formed as a result of the Diels–Alder reaction. The newly developed hydrogel represents a promising platform for further modification in order to obtain programmable wound dressings, patches and drug delivery systems.

## 2. Materials and Methods

### 2.1. Materials

Furfuryl isocyanate (Sigma-Aldrich (Saint Louis, MO, USA), 97%), 6-maleimidohexanoic acid (Sigma-Aldrich, 98%), Benzocaine (Sigma-Aldrich, Pharmaceutical Secondary Standard), sodium periodate (NaIO_4_, Sigma-Aldrich, 99%), N-(3-dimethylaminopropyl)-N-ethylcarbodiimide hydrochloride (EDC, Sigma-Aldrich, 99%), 4-(dimethylamino)pyridine (DMAP, Sigma-Aldrich, 99%), ethanol (Sigma-Aldrich, anhydrous), N,N-dimethylformamide (Sigma-Aldrich), hydroxylamine hydrochloride (NH_2_OH·HCl, Sigma-Aldrich, 99%), hydrochloric acid (HCl, Sigma-Aldrich, 37% aqueous solution), aqueous suspension of cellulose nanocrystals with concentration 10 wt% obtained by sulfuric acid hydrolysis (CNC, University of Maine Process Development) and Hank’s Balanced Salt Solution (HBSS, 1×, without calcium and magnesium ions, Life Technologies, Thermo Fisher Scientific, Carlsbad, CA, USA) were used as received without purification. For dialysis, cellulose-based dialysis bags with a 12 kDa molecular weight cutoff were used.

### 2.2. Synthetic Procedures

#### 2.2.1. Synthesis of Mal-CNC

The aqueous dispersion of CNC was converted to DMF dispersion by dialysis in DMF for 7 days with solvent change twice per day. An amount of 0.528 g of 6-maleimidohexanoic acid (2.50 × mmol) was added to 50 mL of obtained dispersion CNC in DMF with CNC concentration 2 wt% at room temperature and the mixture was cooled down to 0 °C in an ice bath. Then, 0.351 g of DMAP (2.88 mmol) was added at 0 °C. Next, a 10% (*w*/*w*) DMF solution containing 0.388 g of EDC (2.50 mmol) was added dropwise to the reaction mixture and it was stirred at room temperature for 24 h. Obtained Mal-CNC was precipitated in an aqueous acidic solution and then dialyzed against water until neutral pH was achieved with water change twice per day. For determination of the efficiency of modification, the sample was dried first at 60 °C in an oven until powder form was achieved and then at 60 °C in a vacuum oven. The concentration of maleimide moieties was determined by elemental analysis of nitrogen content in the dried sample and was 1.25 mmol/g (yield 50%). The suspension was concentrated to ~10 wt% under rotary evaporation.

#### 2.2.2. Synthesis of Fur-aCNC

Synthesis was performed in two steps. First, aldehyde groups were introduced in CNC by oxidation with sodium periodate (NaIO_4_). Sodium periodate 3.6 g (16.83 mmol) was added to 200 mL of a 1 wt% aqueous suspension of CNC in a 500 mL round bottom flask. The suspension was covered with tin foil to prevent photodecomposition of sodium periodate and stirred for 2 h at room temperature. Then, the suspension was dialyzed against deionized water with water change twice per day. The suspension was concentrated to ~2 wt% under rotary evaporation. For determination of the efficiency of modification, a sample of aCNC was reacted with equimolar (based on theoretical amount of aldehyde groups) amount of hydroxylamine hydrochloride, then dialyzed to remove unreacted products. Then, the sample was dried first at 60 °C in an oven until powder form was achieved and then at 60 °C in a vacuum oven. The concentration of aldehyde moieties was determined by elemental analysis of nitrogen content in the dried sample and was 3.81 mmol/g (yield 90%). Obtained aCNC were converted to DMF dispersion by dialysis in DMF for 7 days with solvent change twice per day. To 50 mL of obtained dispersion, aCNC in DMF with CNC concentration 2 wt% 0.308 g of furfuryl isocyanate (2.50 × mmol) was added at room temperature. Then, the reaction mixture was heated in an oil bath up to 60 °C and stirred for 24 h. Obtained Fur-aCNC was then dialyzed against DMF for 3 days with solvent change twice per day and then dialyzed against water for 7 days with water change twice per day. For determination of the efficiency of modification, the sample was dried first at 60 °C in an oven until powder form was achieved and then at 60 °C in a vacuum oven. The concentration of maleimide moieties was determined by elemental analysis of nitrogen content in the dried sample and was 1.12 mmol/g (yield 45%). The suspension was concentrated to ~10 wt% under rotary evaporation.

#### 2.2.3. Investigation Gelation Conditions

A respective amount of aqueous dispersion of Mal-CNC was mixed with an aqueous dispersion of Fur-aCNC and water to obtain mixtures with a weight ratio of components Mal-CNC/Fur-aCNC = 0.5–2.0 and a total weight concentration (*c_tot_*), from 3 to 10 wt% at 22, 37 and 60 °C. Each system was stirred until a homogeneous mixture was formed (1 min) and left in a water bath for 5 h for complete gelation. The sequence of mixing the reagents had no effect. The assignment of the resulting mixture to a sol or gel state was determined visually using a flip test.

#### 2.2.4. Preparation of Benzocaine-Loaded Hydrogels

Benzocaine (100 mg) was dissolved in 0.5 mL of ethanol. For 2 mL gel formed from equal volume ratios of 7 wt% aqueous dispersion of Fur-aCNC and 7 wt% aqueous dispersion of Mal-CNC, 0.25 mL of benzocaine solution was added. If necessary, the pH of the system was adjusted to 7 by adding HBSS buffer in quantities that did not lead to a change in mass concentrations by more than 0.5 wt%. Gelation was performed for 5 h at 37 °C. The concentration of ethanol was considered to be neglectable.

### 2.3. Characterization

Elemental analysis was carried out using a LECO TruSpec Micro elemental analyzer (CHNS) for samples in powder form (dried one).

Scanning electron microscopy (SEM) was performed at Zeiss Merlin, Zeiss AURIGA at 0.3–1 keV. Samples of CNC, Mal-CNC and Fur-aCNC were used in the form of dispersion diluted to 0.1 wt%, placed on silicon substrate and dried at ambient conditions. The hydrogel sample was prepared by CO_2_ supercritical point drying. The gel sample was soaked for 30 min in 30, 50, 70 and 90% ethanol/water mixtures (*v*/*v*) and then in anhydrous ethanol three times. Ethanol was then removed using an Autosamdri-810 Tousimis critical point dryer and the obtained gel sample was fractured and placed on an SEM grid for analysis.

Image analysis of SEM data was performed in ImageJ (v1.52e). For CNC, Mal-CNC and Fur-aCNC 200 particles were analyzed to obtain their length and diameter; for the gel sample, 120 fibrils were analyzed for the calculation of fibril diameter.

Hydrodynamical radius and zeta potential were determined using a laser particle size analyzer SZ-100 Horiba by dynamic light scattering (DLS).

Rheological properties of the hydrogel were analyzed using a DHR-1 Rheometer (TA Instruments) with a cone geometry (angle is 0.9675°, diameter is 40 mm) with a 27 µm gap. Peltier Plate was used to control the temperature from 22 °C to 60 °C, and a mineral oil was added around the edge of the cone to prevent solvent evaporation. Hydrogel precursors were mixed in a vial and then transferred onto a Peltier plate using a pipette, and then the cone was lowered up to a 27 µm gap and precursors were mixed. The hydrogel precursors were equilibrated at 22 °C, 37 °C or 60 °C for 6 h before experiments.

The mesh size (*ξ*) of hydrogel was determined by *Darcy permeability* as described previously [28]. Hydrogel was formed in a microfluidic chamber fabricated in poly(dimethyl siloxane) with the following geometrical parameters 3 mm × 3 mm × 13.7 mm (width × height × length) (Figure S1, Supplementary Materials). The chamber was connected to an inlet (syringe) and outlet reservoirs filled with water using perfluoroalkoxyalkane tubes and placed in a temperature-controlled incubator. A pressure difference was applied to the hydrogel by variation of the distance (height) between the inlet reservoir relative and the outlet reservoir, in order to achieve pressure-driven water perfusion through the hydrogel. The volumetric flow rate (*Q*) of the water was determined by measuring the change in the mass of the outlet reservoir over time. The Darcy permeability coefficient (*K*) was determined based on Equation (1):

*K* = (*ηLQ*)/*S*∆*P* (1)

where *L* is the hydrogel length (13.7 mm); Δ*P* is the pressure difference across the hydrogel calculated from the difference in height between the inlet at the outlet; *η* is the viscosity of water at 37 °C solution, and *S* is the cross-sectional area of the hydrogel (9 mm^2^). The value of *ξ* for hydrogel was calculated using a previously reported Equation (2) [34]:

*ξ*/2 = [8*K*/(1 − *φ*)]^0.5^ (2)

where *φ* is the volume fraction of the hydrogel.

The swelling ratio (*α*) of hydrogel was investigated at 37 °C in three different media: water with pH 7, HBSS buffer with pH 7 and water with pH 5 (achieved by adding 0.1 M HCl). Hydrogel was weighed and immersed in a glass beaker containing 50 mL of distilled water. After a specific time interval, the hydrogel was taken out and the excess solvent was removed by using filter paper, and the hydrogels were weighed. The swelling index was determined by the following Equation (3):

*α* = (*W_s_* − *W*_0_)/*W*_0_ × 100% (3)

where *Ws* and *W*_0_ are the weights of swollen at a certain time and initial hydrogel, respectively.

The release of benzocaine from the hydrogel specimen was studied in water with pH 7, HBSS buffer with pH 7 and water with pH 5 (achieved by adding 0.1 M HCl). Benzocaine-loaded hydrogels were placed in 100 mL of respective media and kept in the shaking incubator at 37 °C and 100 rpm. First 30 min aliquots (5 mL) were taken every 10 min, second 30 min aliquots (5 mL) were taken every 15 min and after 1 h aliquots (5 mL) were taken every 30 min. After taking each aliquot the same amount of fresh medium was added to make the solution volume constant. Release analysis was carried out for 4 h, and the absorbance of each collected specimen was measured using a CLARIOstar plate reader (BMG LabTech, Offenburg, Germany) at maximum absorbance (λ_max_) 410 nm to quantify the release concentration.

## 3. Results and Discussion

### 3.1. Synthesis of Furan and Maleimide Modified Cellulose Nanocrystals

**Figure 1:**
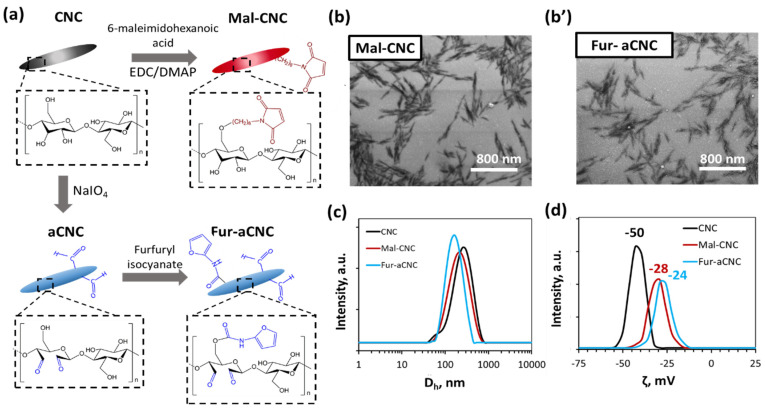
Synthesis and structural parameters of Mal-CNC and Fur-aCNC: (**a**) scheme of synthesis of Mal-CNC and Fur-aCNC; (**b**,**b’**) scanning electron microscopy images of Mal-CNC (**b**) and Fur-aCNC (**b’**); (**c**) the hydrodynamic diameter (*D_h_*) obtained by DLS for CNC, Mal-CNC and Fur-aCNC; (**d**) zeta potential (ζ) for CNC, Mal-CNC and Fur-aCNC.

Cellulose nanocrystals (CNC) were chosen as the basic component of the hydrogel because of their biocompatibility, non-toxicity, rich surface chemistry and the ability to form fibrillar hydrogels with a structure that mimics the structure of living tissues [26]. Two components of the hydrogel based on CNC were synthesized, the first contained a maleimide group (Mal-CNC), and the second contained a furan and aldehyde groups (Fur-aCNC) (Figure 1a). The maleimide group was incorporated according to a previously reported technique [9] consisting of the reaction of CNC dispersed in DMF with 6-maleimidohexanoic acid in the presence of N-(3-dimethylaminopropyl)-N-ethylcarbodiimidehydrochloride/4-(dimethylamino)pyridine (EDC/DMAP) with the formation of an ester bond. The resulting Mal-CNC was converted to aqueous dispersion by dialysis. The content of maleimide groups was determined by elemental analysis of nitrogen and amounted to 1.25 ± 0.20 mmol/g, which corresponds to a reaction yield of 50%.

Modification of CNC by furan fragments took place in two stages: at the first stage, the aqueous dispersion of CNC was oxidized with sodium periodate to introduce aldehyde groups necessary for subsequent interaction with benzocaine; then, the resulting dispersion of aldehyde-modified CNC (aCNC) was converted to DMF dispersion and a reaction with furfuryl isocyanate was carried out by adapting previously reported method [35], leading to the formation of a urethane bond between the aCNC and the functional group. The aldehyde groups were determined by elemental analysis of the reaction derivative with hydroxylamine [36,37], and their content was 3.81 ± 0.45 mmol/g. The content of furan groups was determined by elemental analysis of nitrogen and amounted to 1.12 ± 0.16 mmol/g, which corresponds to the reaction yield of 45%. The low yield of the reaction may be associated with partial degradation of the isocyanate group in the presence of trace amounts of water.

Figure 1b,b’ shows the data of the scanning electron microscopy (SEM) of Mal-CNC and Fur-aCNC. According to the image analysis, the size (length and radius) of CNC has decreased slightly after modification (Figure S2, Supplementary Materials). These data were in agreement with the data of dynamic light scattering (DLS), which showed that the hydrodynamic diameter (*D_h_*) was 182, 176 and 165 nm for CNC, Mal-CNC and Fur-aCNC, respectively (Figure 1c). Modification of CNC affected the zeta potential of the particles. The neat CNC has a potential of −50 mV, while the modified ones have a lower zeta potential (ζ) of −28 and −24 mV for Mal-CNC and Fur-aCNC, respectively (Figure 1d).

### 3.2. Gelation

**Figure 2:**
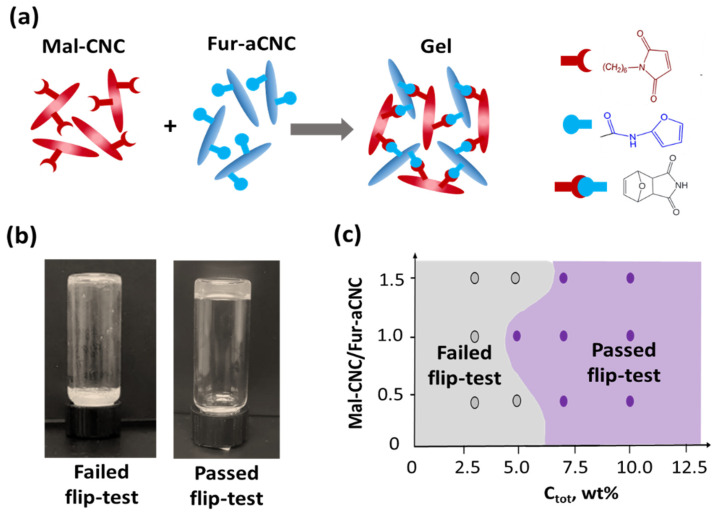
Gelation conditions for Mal-CNC and Fur-aCNC based colloidal system: (**a**) scheme of hydrogel formation via Diels–Alder reaction; (**b**) photographs representing sol state (**left**) and gel state (**right**) of the colloidal system based on Mal-CNC and Fur-aCNC; (**c**) state diagram of the colloidal system based on Mal-CNC and Fur-aCNC.

The state of the colloidal system based on aqueous dispersions of Mal-CNC and Fur-aCNC was studied at various total mass concentrations (*c_tot,_*), component ratios and operating temperatures. The hydrogel is formed due to the Diels–Alder reaction between furan and maleimide groups on the surface of the CNC (Figure 2a). The study of the effect of the total concentration was carried out for the ratio of components 1/1 (which corresponds to approximately the equimolar ratio of functional groups) at room temperature for 6 h. The obtained gel is yellowish, while initial dispersions are colorless. It was found that when the total hydrogel concentration changes from 3 to 10 wt%, gelation, i.e., the formation of a self-supporting system according to the flip test (Figure 2b, right), begins from 5 wt%, and a dense hydrogel that holds its shape is formed at 7 wt% (Figure 2c). Concentration over 10 wt% is difficult to achieve due to the formation of a physical hydrogel between individual particles. The ratio of components affects hydrogel formation—the fastest achievement of the hydrogel point was observed for an equimolar ratio of 1/1, while no hydrogel was formed for a ratio >1/1.5. An increase in the temperature of the experiments to 37 or 60 °C did not change the state of the system (Figure S3, Supplementary Materials). Due to potential bioapplications of designed hydrogel, 37 °C was chosen as the operating temperature.

### 3.3. Rheological Properties and Structure of Hydrogel

**Figure 3:**
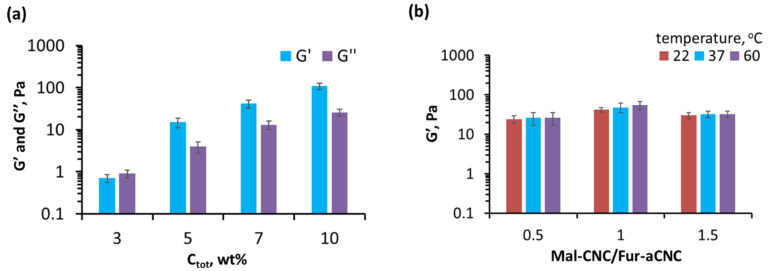
Rheological properties for Mal-CNC and Fur-aCNC based colloidal system: (**a**) variation of storage modulus (*G*′) and loss modulus (*G*″) for hydrogels obtained at 37 °C and the ratio of components Mal-CNC/Fur-aCNC = 1/1, depending on the total concentration of components (*c_tot_*_)_; (**b**) variation of storage modulus (*G*′) for hydrogels *c_tot_* = 7 wt% obtained at 37 and 60 °C depending on variation of the ratio of components Mal-CNC/Fur-aCNC.

Figure 3a shows the change in the storage modulus (*G*′) and loss modulus (*G*″) for hydrogels obtained at 37 °C and the ratio of components Mal-CNC/Fur-aCNC = 1/1, depending on the total concentration of components. Although the system with *c_tot_* = 3 wt% was a gel from a rheological point of view (*G*′ *> G*″) [38] (Figure S4, Supplementary Materials), its mechanical properties were poor (*G*′ = 0.7 ± 0.2 Pa) and it flowed under its own weight (Figure 2b, left) and was marked as sol on the phase diagram in Figure 2c. Systems with *c_tot_* of 5, 7 and 10 wt% demonstrated a storage modulus of 15 ± 4, 42 ± 9 and 109 ± 18 Pa, respectively. Due to the complexity of obtaining highly concentrated initial dispersions with *c_tot_* ≥ 10 wt% and the weak mechanics of the system with *c_tot_* = 5 wt%, the system with *c_tot_* = 7 wt% was selected as the optimal one.

Figure 3b shows the effect of the component ratio on the storage modulus and the reaction temperature. It is shown that an increase in temperature to 60 °C leads to a slight increase in the storage modulus within a relative error of 10%. Thus, rheological studies have shown that the hydrogel storage modulus depends on the total mass concentration and the ratio of components, rather than on the temperature of the reaction. For further investigations, a hydrogel with *c_tot_* = 7 wt% and ratio of components Mal-CNC/Fur-aCNC = 1/1 was chosen as a compromise between the easiness of obtaining and the highest mechanical properties.

**Figure 4:**
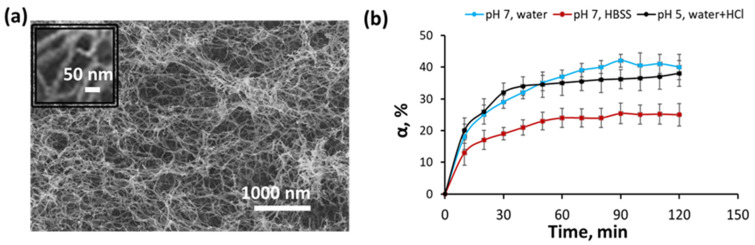
Structure and swelling properties of hydrogel with *c_tot_* = 7 wt% and the ratio of components Mal-CNC/Fur-aCNC = 1/1: (**a**) scanning electron microscopy images of hydrogel with inset demonstrating fibrillar structure in more detail; (**b**) swelling behavior of hydrogel in different media.

The hydrogel structure appears as a random arrangement of fibrils with a diameter of 35 ± 8 nm according to scanning electron microscopy data (Figure 4a). The pore size of the hydrogel was estimated according to the Darcy permeability data and amounted to 250 ± 61 nm (Figure S1, Supplementary Materials). The study of hydrogel swelling in water (pH 7) and HCl aqueous solution (pH = 5) practically did not differ and was characterized by a rapid achievement of equilibrium swelling (about 1 h) and an increase in swelling ratio, up to 50% (Figure 4b). The swelling ratio of hydrogel in HBSS buffer (pH = 7) was smaller in comparison with the swelling ratio in pure water due to the formation of physical bonds between CNC particles as was shown previously [3]. Rather small change in volume during hydrogel swelling is an important advantage for the potential use of hydrogel as the wound dressing or implants.

### 3.4. Release of Benzocaine from Hydrogel

**Figure 5:**
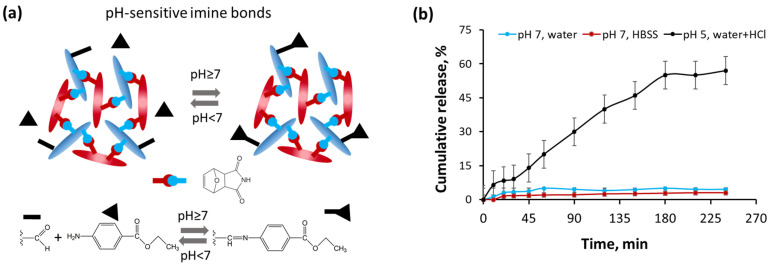
Cumulative release of benzocaine from hydrogel with *c_tot_* = 7 wt% and the ratio of components Mal-CNC/Fur-aCNC = 1/1: (**a**) scheme of pH-dependent formation and hydrolysis of imine bond between the aldehyde groups of the hydrogel and the amino group of benzocaine; (**b**) release rate of benzocaine from hydrogel in different model media.

The advantage of the hydrogel is its ability to form stimuli-responsive bonds with physiologically active substances containing primary amino groups due to the formation of an imine bond. This will allow the utilization of hydrogel for sustained delivery of drugs, including insoluble ones. To study the possibility of the release of physiologically active substances from the gel under different pH, benzocaine was chosen as a model drug. The latter is a well-known local anesthetic and, because it contains a primary amino group, is suitable for conjugation [39]. Figure 5a shows a scheme for the formation of a pH-sensitive imine bond between the aldehyde groups of the hydrogel and the amino group of benzocaine. The formation of an imine bond does not require the use of a catalyst and the only by-product is water. The mild reaction conditions and the absence of toxic by-products make this reaction extremely attractive for bio-applications.

Figure 5b demonstrates the release of benzocaine from the hydrogel at different pH of the medium. In our case, benzocaine is linked to hydrogel by the covalent hydrolyzable bonds, and, therefore, the rate of hydrolysis of the chemical bond is a key factor responsible for the formation of a free drug, whose further release from the hydrogel is controlled by diffusion. Taking into account the similar swelling properties of the hydrogel in the water with pH 7 and 5, the diffusion rate of the free small molecule from the hydrogel should be comparable. It is known that the hydrolysis rate of imine bonds in acidic media is significantly higher than in media with slightly basic and neutral pH [40]. Indeed, a very low release of the substance was observed both in water and the HBSS buffer at pH 7, which corresponds to the data on the stability of the imine bond under these conditions [41]. At the same time, with a decrease in pH due to the addition of HCl, the pH-promoted release of benzocaine was observed. It is worth noting that the release of benzocaine at low pH allows increasing the solubility of benzocaine due to protonation of its amino group, while this drug is poorly soluble at neutral pH [42]. The results obtained are consistent with previously published findings on the release of a drug linked to a polymer forming a hydrogel by an imine bond. For instance, recently Mahapatra et al. reported the development of the acrylamide/PEI hydrogel containing imine-linked ampicillin [43]. Enhanced drug release at pH 5, compared to pH 7, was observed for hydrogels with different drug loading.

## 4. Conclusions

In this study, a fibrillar colloidal hydrogel formed from anisotropic rod-shaped cellulose nanocrystals covalently crosslinked via the Diels–Alder reaction was developed. Although the hydrogels crosslinked by the Diels–Alder reaction have been previously reported [21], we proposed and studied for the first time hydrogels consisting only of nanoparticles, while previous examples were based on the nanoparticle–polymer or solely polymer systems. The advantage of a nanoparticle-based system is improved permeability properties (higher mesh size) and preservation of the fibrillar structure for any ratio of components. In contrast, entirely polymeric systems exhibit no fibrillarity at all, while the fibrillarity of nanoparticle–polymer systems is strongly dependent on the ratio of components. In our case, due to the utilization of CNCs, the fibrillar structure of the resulting hydrogel was achieved. This structural feature allows approximating mechanical and transport properties of the hydrogel material to living systems, which in many cases have a fibrillar nature [26].

The introduction of aldehyde groups into the hydrogel led us to achieve a pH-sensitive programmable release of benzocaine, which was successfully released at pH~5 due to the acidic-promoted hydrolytic cleavage of the imine bond formed between the aldehyde groups of the hydrogel and the amino group of benzocaine. Moreover, under conditions of pH-sensitive release, hydrogel degradation did not occur, since the gel skeleton was formed by crosslinking based on Diels–Alder reaction adducts, which allows substances to be loaded into the gel repeatedly. Thus, the developed colloidal hydrogels can become a simple and convenient platform for creating wound dressings with a pH-sensitive release of physiologically active substances containing an amino group.

## Data Availability

The data presented in this study are available on request from the corresponding author.

## Supplementary Materials

The following supporting information can be downloaded at: https://www.mdpi.com/article/10.3390/polym15244689/s1, Figure S1: details of experiments on Darcy’s permeability; Figure S2: geometrical dimensions of CNC; Figure S3: state diagram of the colloidal system based on Mal-CNC and Fur-aCNC at 37 and 60 °C; Figure S4: rheological behavior of system with c_tot_ = 7 wt% obtained at 37 °C and ratio of components Mal-CNC/Fur-aCNC = 1; References [44,45] are cited in the supplementary materials.
