# Supplementary material for: Fibrillar Hydrogel Based on Cellulose Nanocrystals Crosslinked via Diels–Alder Reaction: Preparation and pH-Sensitive Release of Benzocaine
Source: Polymers (Basel). 2023 Dec 13;15(24):4689. doi: 10.3390/polym15244689 (PMC10748274; doi:10.3390/polym15244689)
Supplement: Supplementary file 1 [file polymers-15-04689-s001.zip › polymers-2765208-supplementary.pdf]

Supplementary information

Fibrillar Hydrogel Based on Cellulose Nanocrystals Crosslinked via Diels-Alder Reaction: Preparation and pH-Sensitive Release of Benzocaine

Sofia M. Morozova [1]*, Evgenia G. Korzhikova-Vlakh [2]

1 N.E. Bauman Moscow State Technical University, 2nd Baumanskaya St. 5/1, 105005 Moscow, Russian Federation
2 Institute of Macromolecular Compounds of Russian Academy of Sciences, Bolshoy pr. 31, 199004, St. Petersburg, Russia; vlakh@hq.macro.ru (E.K.-V.)
* Correspondence: sofiionova@yandex.ru (S.M.), Tel. +79859108502

S1. Darcy permeability measurements

Figure S1a demonstrates appearance of the microfluidic device used for Darcy's permeability measurements as previously described.[1][2] Each chamber has parameters 3 mm × 3 mm × 13.7 mm (width × height × length). Device was fabricated using poly(dimethyl siloxane). **Figure S1b** shows design of the experiment: preheated reservour with media was placed at certain height (to have linear dependence of volumetric flow (Q) from pressure difference (ΔP)). Media was diffused through hydrogel under gravitation and was collected in outlet reservour and weighted.

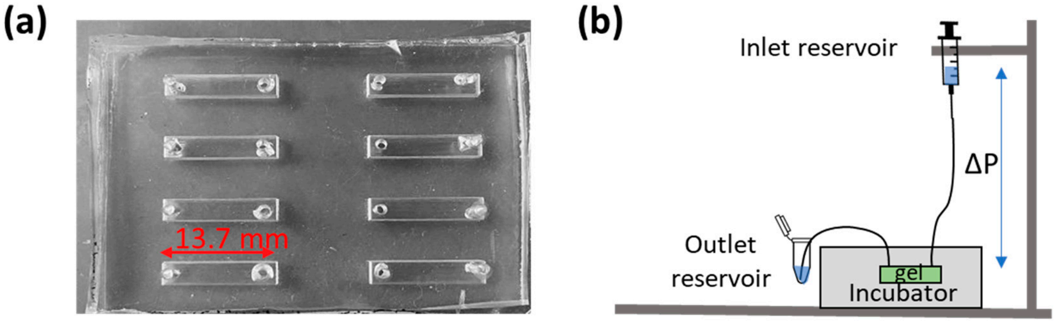

Figure S1. Details of experiments on Darcy's permeability. (a) Appearance of microfluidic device for Darcy's permeability measurements. (b) Design of the experiment for Darcy's permeability measurements.

S2. Nanoparticle characterization.

Figure S2a,b demonstrated Length (l) and diameter (d) of cellulose nanocrystals (CNC) before and after modification. Data was obtained by image analysis of scanning electron microsopy (SEM) images of CNC, maleimide functionalized CNC (Mal-CNC), and furyl and aldehyde functionalized CNC (Fur-aCNC). The diameter of CNC, Mal-CNC and Fur-aCNC was 16±4 nm, 15 ± 5 nm and 15± 6 nm, respectively, while their length was 177 ± 36 nm, 170 ± 41 nm and 154 ± 45 nm, respectively. Reduction of the size in case Fur-aCNC is in agreement with previously reported data about oxidation of CNC with respective concentration of sodium periodate.[3]

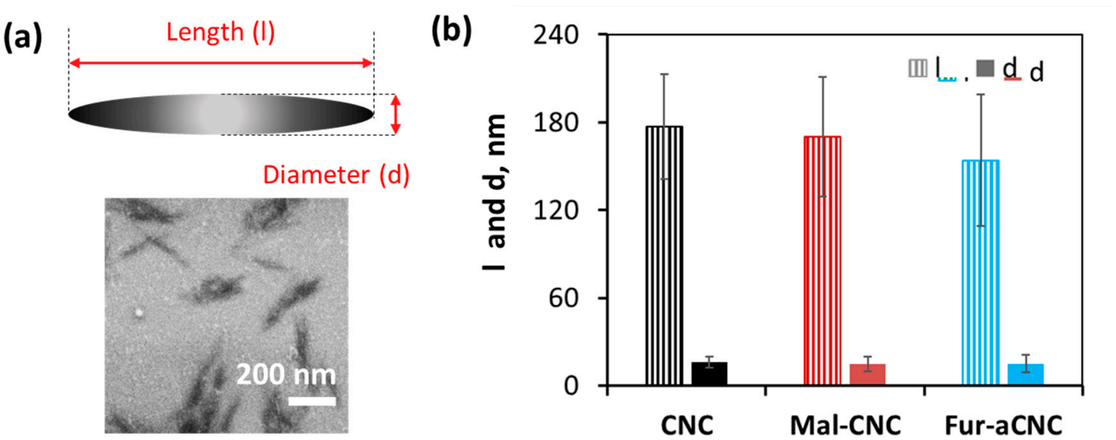

Figure S2. Geometrical dimensions of CNC. (a) Scheme of CNC geometrical parameters and scanning electron image of Fur-aCNC. (b) Length (l) and diameter (d) of cellulose nanocrystals (CNC) before and after modification for CNC, Mal-CNC and Fur-aCNC based on SEM image analysis.

S3. Gelation of Mal-CNC and Fur-aCNC

Gelation of Mal-CNC and Fur-aCNC was investigated at different temperatures, namely, 22, 37 and 60 oC. Two types of system states were indicated: passing flip test (when system is a self-supporting gel) and failed flip test (when system flow). Diagrams at all tempearture were identically and presented on Fig. 2c in main text (22 °C) and in Figure S3a,b for 37 and 60 °C, respectively.

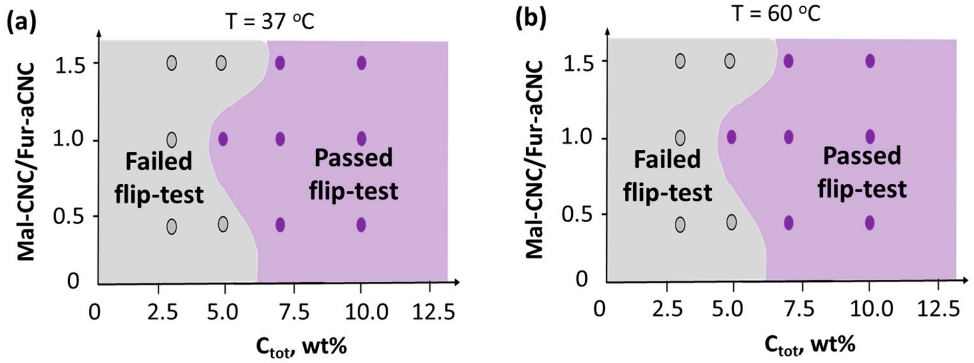

Figure S3. State diagram of the colloidal system based on Mal-CNC and Fur-aCNC at 37 (a) and 60 °C (b).

S4. Rheolgical behaviour of gels

Figure S4 presents rheological behaviour of system with c_{tot} = 7 wt.% obtained at 37 °C and ratio of components Mal-CNC/Fur-aCNC=1. Gel point (when G'>G'') was achieved in ~20 minutes. However, due to low mechanical properties this system flow under it's own weight.

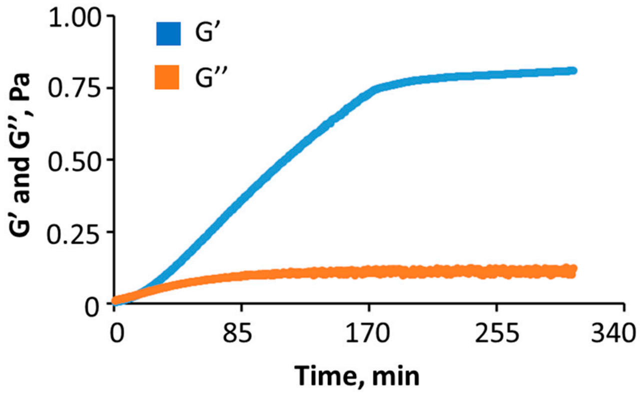

Figure S4. Rheological behaviour of system with c_{tot} = 7 wt.% obtained at 37 ^{o}C and ratio of components Mal-CNC/Fur-aCNC=1.

References

[1] E. Huang, Y., Morozova, S. M., Li, T., Li, S., Naguib, H. E., & Kumacheva, "Stimulus-Responsive Transport Properties of Nanocolloidal Hydrogels," *Biomacromolecules*, vol. 24, no. 3, pp. 1173–1183, 2023, doi: 10.1021/acs.biomac.2c01222.

[2] N. Khuu, M. Alizadehgiashi, A. Gevorkian, E. Galati, N. Yan, and E. Kumacheva, "Temperature-Mediated Microfluidic Extrusion of Structurally Anisotropic Hydrogels," *Adv. Mater. Technol.*, vol. 4, no. 6, pp. 1–9, 2019, doi: 10.1002/admt.201800627.

[3] X. Tian and X. Jiang, "Preparing water-soluble 2 , 3-dialdehyde cellulose as a bio- origin cross-linker of chitosan," *Cellulose*, vol. 25, pp. 987–998, 2018, doi: 10.1007/s10570-017-1607-0.
